# MicroRNA-152 and -181a participate in human dermal fibroblasts senescence acting on cell adhesion and remodeling of the extra-cellular matrix

**DOI:** 10.18632/aging.100508

**Published:** 2012-12-09

**Authors:** Mara Mancini, Gaelle Saintigny, Christian Mahé, Margherita Annicchiarico-Petruzzelli, Gerry Melino, Eleonora Candi

**Affiliations:** ^1^ University of “Tor Vergata”, Department of Experimental Medicine and Surgery, 00133 Rome, Italy; ^2^ CHANEL Parfums Beauté, F- 92521, Neuilly/Seine, France; ^3^ IDI-IRCCS, Laboratory of Biochemistry, c/o Department of Experimental Medicine and Surgery, University of “Tor Vergata”, 00133 Rome, Italy; ^4^ Association Cell Death & Differentiation, c/o Department of Experimental Medicine and Surgery, University of “Tor Vergata”, 00133 Rome, Italy; ^5^ Medical Research Council, Toxicology Unit, Hodgkin Building, Leicester University, Leicester LE1 9HN, UK

**Keywords:** aging, skin, miRNA, MIR, senescence

## Abstract

Ageing of human skin is associated with phenotypic changes in the cutaneous cells; the major functional markers of ageing occur as consequences of dermal and epidermal cell senescence and of structural and compositional remodeling of normally long-lived dermal extracellular matrix proteins. Understanding the contribution of the dermal cells in skin ageing is a key question, since this tissue is particularly important for skin integrity and its properties can affect the epidermis. Several microRNAs have been shown to be involved in the regulation of pathways involved in cellular senescence and exerted important effects on tissues ageing. In this study, we demonstrate that the expression of miR-152 and miR-181a increased during the human dermal fibroblasts senescence and that their overexpression, is sufficient to induce cellular senescence in early-passage cells. The increase of these miRNAs during cells senescence was accompanied by a decrease in integrin α and collagen XVI expression at mRNA and/or protein levels resulting in reduced cellular adhesion and suggesting extracellular matrix remodeling. These findings indicate that changes in miRNAs expression, by modulating the levels of adhesion proteins and extra-cellular matrix components, such as integrin α and collagen XVI, could contribute to the compositional remodelling of the dermis and epidermis occurring during skin aging.

## INTRODUCTION

Senescence is a biological state in which the cells have an irreversible proliferative arrest while they continue to be metabolic active. The senescent phenotype is acquired in vitro after multiple rounds of cell division (replicative senescence) or upon oncogenes activation or oxidative stress (stress-induced premature senescence) [1-8]. The mechanisms underlying replicative senescence include telomere shortening, permanent growth arrest from G1 to S phase of the cell cycle, enhanced beta-galactosidase activity and increased expression of key mediators including p53, promyelocitic leukemia protein (PML), p16INK4a and p19Arf [1,9-12]. While cellular senescence is considered a protective mechanism against cancer, it has also been hypothesized that the progressive accumulation of senescent cells in some tissues may contribute to several age-related diseases and organismal aging [13-15]. Progressive DNA damage and mitochondrial decline are both considered to be prime instigators of natural ageing. These two pathways have been viewed largely in isolation. However, recent studies have revealed a molecular circuit that directly links DNA damage to compromised mitochondrial biogenesis and function via p53 [16-22]. Furthermore, the mitochondrial function decline is considered a major mechanism underlying senescence. Damaged mitochondria not only produce less ATP but also generate increased amounts of reactive oxygen species and display a greater propensity to trigger apoptosis [23-29].

The aging process, especially of the skin, is governed by changes in the epidermal, dermo-epidermal and dermal compartments. Skin function is mediated primarily by the structure of the epidermal and dermal layers [30,31]. The highly cellular, yet avascular, epidermis forms a barrier, which prevents both water and heat loss and the entry of pathogenic organisms. In contrast, the dermis is both vascularised and relatively acellular. The two layers are joined by a compositionally complex undulating dermo-epidermal junction (DEJ) in which basal epidermal keratinocytes are secured to a type IV collagen-rich basement membrane by hemidesmosomes and the dermis is anchored by collagen VII fibrils and fibrillin-rich microfibril bundles [32-36]. Sparsely distributed, dermal fibroblasts are thought to be responsible for synthesizing the three major groups of dermal extracellular matrix (ECM) proteins and collectively these ECM assemblies not only dominate the structure and function of the dermis, but via aberrant remodeling, mediate the changing function of aging skin. Aged skin is characterized by a flattening of the dermo-epidermal junction, a marked atrophy and a loss of elasticity of the dermal connective tissue [37], associated with a reduction and disorganization of its major extracellular matrix components, such as collagen and other elastic fibers [38], proteoglycans and glycosaminoglycans [39]. Some studies proposed that the consequences of ECM changes affected fibroblast phenotypic behavior in the dermis asserting that this cell situated in a damaged matrix were no longer subjected to mechanical stretching via integrin-collagen interactions [40-42]. Alteration of fibroblast properties, associated with aging or senescence, have been studied either in long-term cell cultures [43-48], or by using fibroblasts from skin biopsies derived from donors with increasing age [49]. The observed age related changes in fibroblast include cell morphology, metabolism [50], decline in the production of extracellular matrix proteins such as type I and III collagens [41], and overexpression of proteases involved in the degradation of the extracellular matrix [51]. All senescing cells undergo profound changes in gene expression. Altered gene expression gives rise to the senescent phenotype, and is well established as part of the mechanisms and pathways that activate the senescence program in cells. However, the factors responsible for the alterations of gene expression during senescence remain elusive.

MicroRNAs (miRNAs) are key modulators of gene expression in various biological and pathological processes. These RNAs, ~22 nucleotides in size, act as sequence guides that direct Argonaute protein complexes to mRNAs, where they decrease protein synthesis through translational repression or mRNA degradation and thereby influence many basic cellular processes and diseases [52-58]. Changes in miRNA expression levels occur in cellular senescence and organismal aging [59-64], and have been linked to changes in levels of mRNAs that are putative targets of specific miRNAs [65-67]. The regulation of miRNAs expression during senescence and aging represents an emerging and the list of miRNAs and corresponding target mRNAs involved in these pathways is rapidly increasing [68-73]. Recently, in a model of in vitro replicative senescence of normal human epidermal keratinocytes neonatal (HEKn), we have identified miR-191 as an anti-proliferative and senescence-associated miRNA [74] and also a ΔNp63α-miRNAs regulatory loop that represents a “stemness master gene”-mediated strategy to promote proliferation and to counteract senescence [75]. Several studies have identified specific sets of miRNAs up-regulated in keratinocytes and fibroblasts replicative senescence [75-78], among these we focused on miR-152 and miR-181a. MiR-152, although has been detected in replicative-induced fibroblast senescence screenings [76], has never been studied to identify its targets and its role in the senescence process. MiR-181a expression is up-regulated during keratinocytes replicative senescence and its overexpression is sufficient *per se* to induce cellular senescence [75].

Here, we have investigated the role of miR-152 and miR-181a in replicative senescence of primary human dermal fibroblasts (HDFn) providing evidence that the expression of these miRNAs in young proliferating fibroblasts is sufficient to induce senescence markers. We have also identified as new targets involved in this process, itga5 and col16a1, whose decrease is probably involved in ECM aberrant remodelling of aged skin.

## RESULTS

### Induction of replicative senescence in human dermal fibroblasts

**Figure 1:**
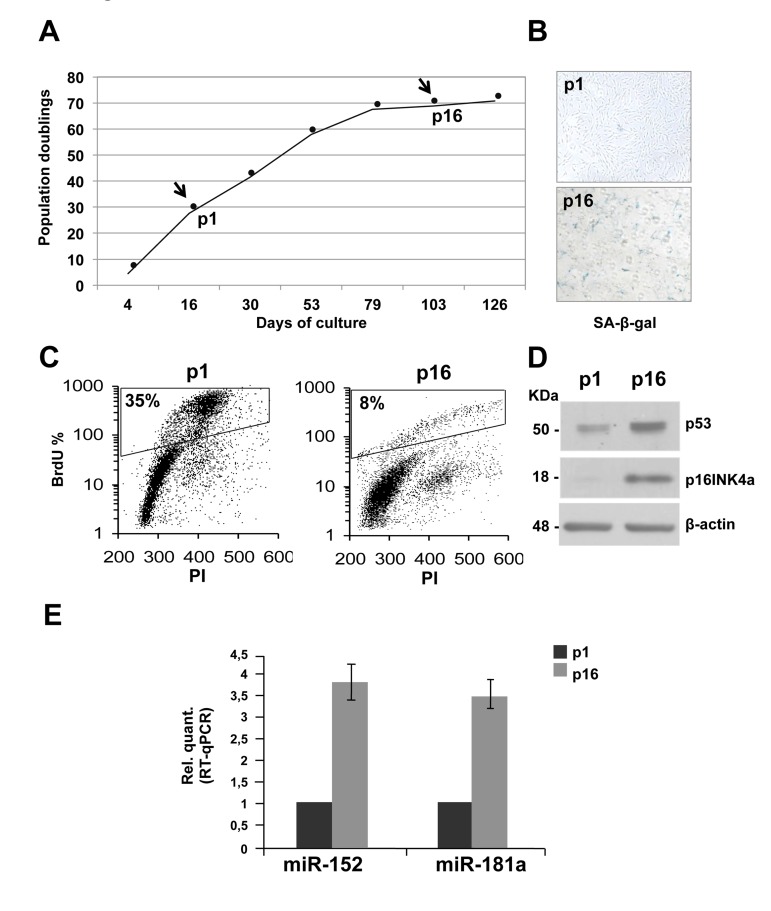
Replicative senescence in human dermal fibroblasts (HDFn) **(A)** Growth curve of cultured HDFn. **(B)** SA-β-gal staining of HDFn cells at p1 and p16 in culture. **(C)** HDFn cultured cells at p1 and p16 (p, passage) were subjected to a 4h BrdU-pulse, then collected, PI stained and analyzed by flow cytometry. BrdU positive cells are indicated as S phase fluorescent population and are assessed by PI staining of DNA content of 2n or 4n (fixed to values of 200 and 400 in the plots). **(D)** Western blots performed on proteins extracts from HDFn at p1 and p16 showing the analysis of some senescence markers, such as p53 and p16 levels. β-actin was used as loading control. **(E)** Real Time RT-qPCR was employed to analyze the expression levels of miR-152 and miR-181a in HDFn cells at a growing number of passages in culture (p1-p16). Values reported are the average ± SD of three independent experiments.

To generate a model of replicative senescence in which to assess miRNAs expression profile, proliferating primary HDFn cells were monitored through serial passaging. The proliferating or senescent state of the cells was assessed morphologically. A growth curve that calculated the population doublings at each passage was generated (Figure 1A). After sixteen passages (p), the proliferation rate slows down, leading to a complete block after seventeen passages. As shown in figure 1B, at p16 senescent cells stained positive for SA-β-galactoside compared to the proliferating population at p1. Between p1 and p16 we also observed a significant decrease in the percent of BrdU incorporating cells (from 35% to 8%, Figure 1C) indicating cell cycle G1-arrest. Finally, as further control, a western blot analysis was performed on protein extracts from HDFn cells collected at p1 and p16 passages. As expected, p53 and p16INK4a, which are senescence markers, are induced during passages (Figure 1D). In a previous study Yong Wang and colleagues showed, using microarray approach, that miR-152 is among the miRNAs more upregulated in replicative-induced fibroblast senescence [76] but it has never been studied to identify its targets and its role in the senescence process. In addition, by in silico analyses we found that miR-152 and miR-181a, the latter involved in keratinocytes-induced senescence [75], have as putative targets many mRNA involved in cell adhesion and extracellular matrix remodelling, therefore we decide to study the role of these miRNAs in our experimental system. MiRNAs expression levels were assessed and compared, by Real Time-qPCR, in RNA samples purified from proliferating (p1) and senescent (p16) HDFn. We confirmed, by real-time PCR that both, miR-152 and miR-181a, are significantly upregulated during fibroblasts replicative-induced senescence (Figure 1E).

### miR-152 and miR-181a induce senescence in human dermal fibroblasts

**Figure 2:**
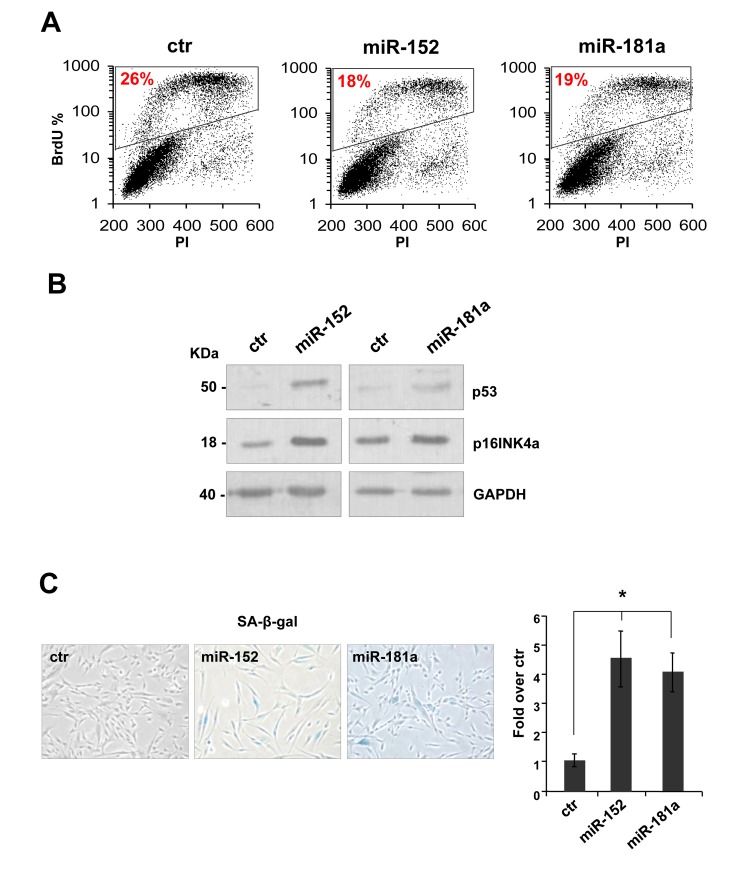
miR-152 and miR-181a induce cellular senescence in HDFn cells **(A)** 96 h after transfection of HDFn cells with scramble control (Ctr), miR-152 or miR-181a sequence, cells were subjected to a 4h BrdU-pulse, then collected, PI stained and analyzed by flow cytometry as described in Figure 1. **(B)** Western blot analysis of protein extract of HDFn transfected with miR-152 and miR-181a versus scramble control sequence (ctr). MiR-152and miR-181a overexpression increase p53 and p16INK4 protein level. β-actin was used as loading control. **(C-D)** SA-β-gal staining and quantification by blue cells counting/field (as fold over control). Values reported are average ± SD of three independent stains. *p-Value <0.01 by Student's t test.

To investigate the role of miR-152 and miR-181a in human dermal fibroblast, we set up overexpression by transfection with pre-miR-152, pre-miR-181a, or scrambled control sequence in proliferating HDFn cells. The miRNAs were overexpressed 3-5 folds to mimic their physiological expression level during senescence. Ninety-six hours after transfection we observed a decrease about 40% in BrdU incorporation in the presence of miR-152 and miR-181a (respectively 18% and 19% versus 26% of scrambled control, Figure 1A) indicating a cell cycle G1-arrest. Overexpression of miR-152 and miR-181a had additional effects in HDFn cells, including the induction of the senescence markers p53 and p16INK4a(Figure 2B) and an increase in the number of cells positive for SA-β-galactosidase staining as shown by the images and blue cell quantification in Figure 2C and 2D. These results suggest that miR-152 and -181a overexpression, is sufficient *per se* to induce cellular senescence.

### miR-152 represses ITGA5 expression and controls HDFn cell adhesion

**Figure 3:**
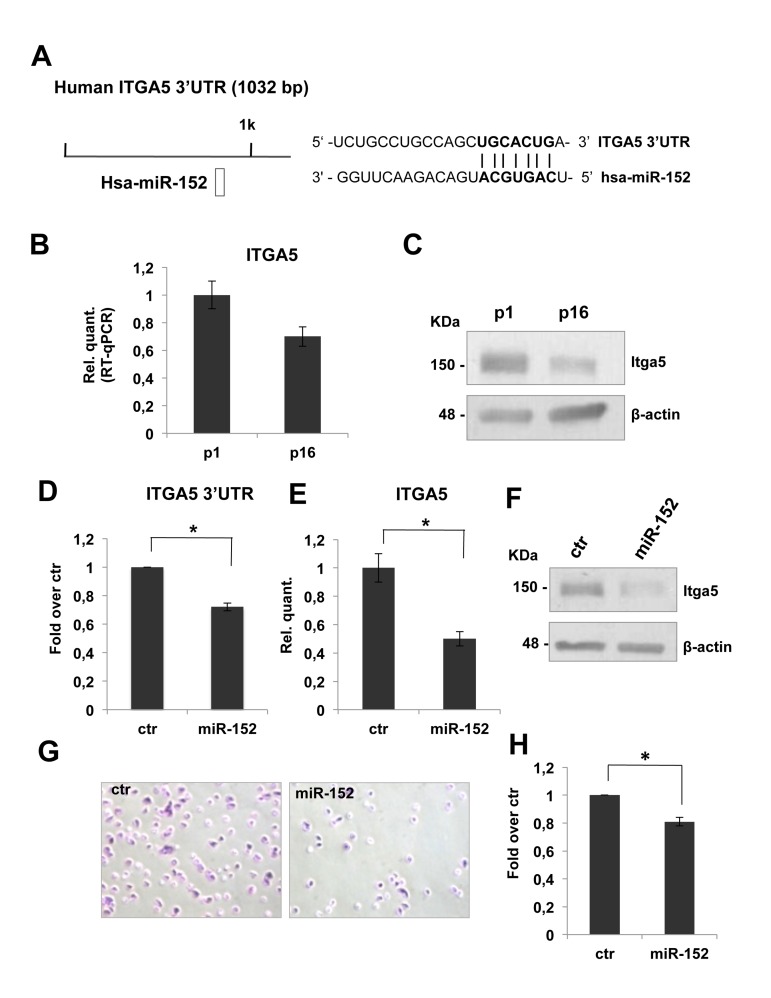
miR-152 represses ITGA5 expression and controls HDFn cell adhesion **(A)** Predicted miR-152 target sites on human ITGA5 3'UTR were identified by TargetScan 6.2 software. **(B)** Real Time RT-qPCR was employed to analyze the expression level of ITGA5 in HDFn cells at a growing number of passages in culture (p1-p16). Values reported are the average ± SD of three independent experiment. **(C)** Western blot of HDFn cells protein extracts collected at increasing passage number in culture (p1-p16). Itga5 protein level is shown and β-actin was used as loading control. **(D)** Insertion of ITGA5 3'UTR target sequence in a luciferase reporter vector leads to diminished luciferase activity in presence of miR-152 in HEK293 cells 24h after co-transfection. Histograms show the values resulting as the average ± SD from three independent co-transfections.**(E)** Real Time RT-qPCR was employed to analyze the expression level of ITGA5 in proliferating HDFn cells transfected with miR-152 versus a scrambled control sequence (Ctr). Values reported are the average ± SD of three independent experiment. **(F)** Western blot analysis of protein extracts of HDFn transfected with miR-152 versus a scrambled control sequence (Ctr). miR-152 overexpression decreases ITGA5 protein levels; β-actin was used as a loading control. **(G)** Adhesion assay performed on proliferating HDFn cells transfetcted with miR-152 versus scrambled control sequence. **(H)** Histogram shows adhesion ability of proliferating HDFn 96h after transfection with control or miR-152. Values reported are the average ± SD of three independent experiment. *p-Value <0.01 by Student's t test.

To investigate the role of miR-152 on ECM remodeling, we started a detailed analysis of its putative molecular targets. From an *in silico* prediction, by TargetScan 6.2 database, we selected ITGA5-3'UTR as putative target of miR-152. These 3'UTR harbors at least one miR-152 target site that is highly conserved among vertebrates (Figure 3A). As shown in figure 3B, real time-qPCR analysis showed a significant decrease (about 30%) of ITGA5 mRNA level between p1 and p16 fibroblasts. The down-regulation of miR-152 putative target, was also possible confirmed at the protein level, in fact a western blot analysis showed a decrease of itga5 protein level in HDFn cells from p1 to p16 (Figure 3C). The observed inverse correlation between miR-152 expression and its putative target strengthened the hypothesis of its direct regulation. Integrins consist of 18 α- and 8 β-glycoprotein subunits, which form 24 distinct heterodimeric transmembrane receptors. These receptors bind to ECM proteins such as fibronectin to transport signals bidirectional across the cell membrane, allowing cells to respond to environmental changes [79,80]. Furthermore, integrin binding to ECM is not only required for transducing signals from the matrix to cells, but this interaction also initiates responses that allow the cells to organize and remodel the matrix [81], this features is very limited in aged tissues and in particular in aged skin. The mesenchymal integrins α5 (ITGA5) and β1 form heterodimers to mediate cell adhesion to fibronectin [82]. Knock-out of ITGA5 in mice results in embryonic lethality [83]. In human epatocarcinoma cells, ITGA5 promotes cell adhesion and migration on fibronectin through activating focal adhesion kinase [84]. This findings indicate that ITGA5 expression plays an important role to enhance cell adhesion to and migration on fibronectin.

To confirm that ITGA5 mRNA is direct miR-152 target, we cloned ITGA53'UTR sequence, containing the miR-152 conserved binding site, downstream of a luciferase reporter gene. Relative luciferase activity, quantified 24 h after the transfection of reporter construct in presence of pre-miR-152, demonstrated that miR-152 repressed luciferase activity controlled by ITGA5 3-UTR (Figure 3D). Moreover miR-152 transfection in proliferating HDFn is followed by a significant decrease (about 50%, Figure 3E) of ITGA5 mRNA level and led to a strong down-regulation of itga5 protein level (Figure 3F) demonstrating that miR-152 was able to repress HDFn itga5 endogenous expression.

To define the role of miR-152 and its molecular target ITGA5 in HDFn cell adhesion, we tested cell adhesion of proliferating HDFn over-expressing miR-152 compared to scrambled control. We found a decrease of about 20% in cells adhesion in miR-152 transfected cells as compared to control as shown in Figure 3G-H. These results suggested that miR-152 contribute to fibroblast adhesion in part by down regulation of ITGA5.

### COL16A1 expression is downregulated in senescent HDFn cells and its 3'-UTR is a direct miR-181a target

To identify potential miR-181a putative targets, we used the TargetScan 6.2 database. COL16A1 was the best candidate target to investigate the possible role of miR-181a on ECM remodeling. Collagen XVI is a member of the fibril associated collagens with interrupted triple helices (FACIT) and constitutes a minor component of the skin ECM. The presence of collagen XVI in the DEJ zone of the papillary dermis indicates an active role in anchoring microfibrils to basement membranes. By interconnecting ECM proteins to cells, collagen XVI is likely to be able to move these proteins and hence affects ECM networks, ensuring mechanical anchorage of the cell and outside-inside signal transduction [85-86].

**Figure 4:**
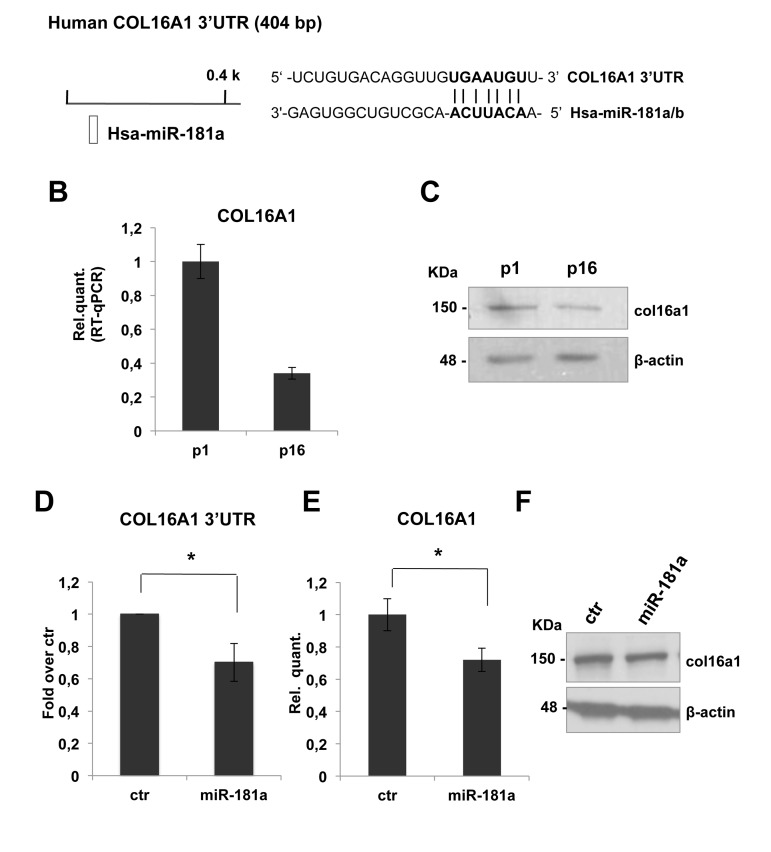
COL16A1 expression is downregulated in senescent HDFn cells and its 3'-UTR is direct miR-181a target **(A)** Predicted miR-181a target site on human COL16A1 3'UTR was identified by TargetScan 6.2 software. **(B)** Real Time RT-qPCR was employed to analyze the expression level of COL16A1 in HDFn cells at a growing number of passages in culture (p1-p16). Values reported are the average ± SD of three independent experiment. **(C)** Western blot of HDFn cells protein extracts collected at increasing passage number in culture (p1-p16). Col16a1 protein level is shown and β-actin was used as loading control. **(D)** Insertion of COL16A1 3'UTR target sequence in a luciferase reporter vector leads to diminished luciferase activity in presence of miR-181a in HEK293 cells 24h after co-transfection. Histograms show the values resulting as the average ± SD from three independent co-transfections. **(E)** Real Time RT-qPCR was employed to analyze the expression level of COL16A1 in proliferating HDFn cells transfected with miR-181a versus a scrambled control sequence (ctr). Values reported are the average ± SD of three independent experiment. **(F)** Western blot analysis of protein extracts of HDFn transfected with miR-181a versus a scrambled control sequence (ctr). Itga5 protein levels is shown and β-actin was used as a loading control.

COL16A1-3'UTR harbours a putative target sequence for miR-181a that is highly conserved among vertebrates (Figure 4A). As shown in figures 4B and 4C, real time-qPCR showed a decrease of COL16A1 mRNA and protein level in dermal fibroblasts from p1 to p16. This inverse correlation with miR-181a expression is consistent with the hypothesis that they might regulate COL16A1. To confirm these, we cloned COL16A1-3'UTR sequence downstream of luciferase cDNA, to use them in luc-reporter assays. Transfecting COL16A1-3'UTR reporter construct in presence of the pre-miR-181a or scrambled sequence, we obtained a significant downregulation, about 30%, in relative luciferase activity (Figure 4D). Overexpression of miR-181a by transfection in proliferating HDFn led to a significant decrease in a COL16A1 mRNA (about 30%, Figure 4E) but not in protein level as shown by western blot analysis in Figure 4F. These results suggested that COL16A1 mRNA is a direct target of miR-181a, we think that we could not detect the decrease at protein level, upon 96h transient transfection, because col16a1 is a very stable protein.

## DISCUSSION

Here, we have identified two miRNAs, miR-152 and miR-181a, upregulated in senescent human diploid fibroblasts. We have also shown that 3-5 fold expression of the two miRNAs is sufficient *per se* to induce senescence markers (SA-*β-gal* staining, reduced cell proliferation and expression of p16INK4a) in proliferating, young fibroblasts. We have identified ITGA5 as direct targets of miR-152. Integrins, beside transducing signals from the matrix to cells, bind to ECM to allow the fibroblasts to organize and remodel the matrix [81], this features is very limited in aged tissues and in particular in aged skin and this could be due in part to ITGA5 downregulation mediated by miR-152. In addition, we have shown that overexpression of miR-152 in fibroblasts significantly reduced cell adhesion, this finding indicate that ITGA5 might have a role in aged skin tissue. On the other hand, miR-181a target is COL16A1 mRNA, collagen XVI is a minor component of the skin ECM, nevertheless it is expressed in the DEJ zone of the papillary dermis and it connects ECM proteins to cells, ensuring mechanical anchorage of the cell and outside-inside signal transduction [85, 86]. Overall, these findings suggest a model whereby during replicative senescence, a set of miRNAs, among them miR-152 and miR-181a, are upregulated, this sustain the senescent phenotype. The mechanisms through which the miRNAs are upregulated will deserve further investigation. Although we do not exclude the possibility that other important senescence-associated miRNA-152 and miR-181a targets play a role in the senescent phenotype observed, we believe that the reduction in expression of ITGA5 and COL16A1 by miR-152 and miR-181a, strongly suggests that miRNAs have a complex role also in ECM remodeling typical of aged skin that deserve to be further investigated.

## MATERIAL AND METHODS

### Cell culture and Transfection

Human Dermal Fibroblasts neonatal (HDFn, Cascade, Invitrogen, Carlsbad, California, USA) were cultured in 106 medium added with LSGS growth supplements (Cascade). Cells were passaged usually once a week, at each passage the harvested cells number and seeded cell number were recorded in order to calculate the population doublings occurring between passages and the population doubling time. At each passage different aliquots of the cells were harvested to extract in triplicate RNA and proteins and an aliquot was submitted to senescence activated β-galactosidase staining in order to assay the senescent or non-senescent state of the cells. Human primary fibroblasts were transfected with human pre-miR 181a, pre-miR-152, and scramble sequence as negative control (Ambion, Texas, USA) using the Lipofecatmine RNAimax transfection reagent (Invitrogen) according to manufacturer protocols. 24hrs after transfection, the medium was removed and replaced with fresh medium.

### RNA Extraction and Real Time PCR analysis

Total RNA from cells was isolated using mirVana mirRNA Isolation Kit (Ambion) following the manufacturer's protocol. Total RNA was quantified using a NanoDrop Spectophotometer (Thermo Scientific, Delaware, USA) and RNA quality was controlled on an 1% agarose gel. For microRNA detection, RNA was reverse transcribed using TaqMan MicroRNA Reverse Transcription kit and qRT-PCR was performed with TaqMan universal master mix (Applied Biosystem) and specific primers for miR-181a and miR-152. U18 was used as housekeeping gene for normalization (Applied Biosystem).

For determination of mRNA expression level, total RNA was reverse transcribed with GoScriptTM Reverse Transcription System (Promega, Madison, WI, USA) according to manufacturer's protocols. Real Time PCR was than performed with COL16A1 or ITGA5 specific primers by using the Platinum SYBR Green qPCR SuperMix UDG (Invitrogen). The sequences of the primers used in this study were as follow: hCOL16A1F 5' –CTTCAACCTCATCCACCGACTCAG- 3', hCOL 16A1R 5' –TAGTGTCAGCACCAGGGCGGCAAAC TC- 3', hITGA5F 5'-TTGTTGCTGGTGTGCCCAAA G-3', hITGA5R 5'-GCCATCTGTTCCCCTGAGAAG-3'. β-Actin was used as a housekeeping gene for normalization. The expression of each gene and miR was defined from the threshold cycle (Ct), and relative expression levels were calculated by using the 2^−ΔΔCt^ method after normalization with reference to expression of housekeeping genes.

### Senescence-Associated β-Galactosidase Staining

Cells were grown in 6-well culture plates, washed with PBS, and fixed with 2% formaldehyde/0.2% glutaral-dehyde/2mM MgCl_2_ in PBS for 5 minutes. After another washing step with PBS, cells were incubated with β-galactosidase staining solution (2 mM MgCl_2_, 5 mM potassium ferricyanide, 5 mM potassium ferrocyanide, 1 mg/mL 5-bromo-4-chloro-3-indolyl- β-Dgalactoside [X-gal], pH 6.0) for 24 hours at 37°C. The reaction was stopped by replacing the staining solution with 70% glycerol.

### Cell proliferation and cell cycle analysis

Incorporation of bromodeoxyuridine (BrdU) during DNA synthesis was evaluated with the Click-iT™ EdU flow cytometry assay kit, following the manufacturer's protocol (Molecular Probes, Eugene, OR, USA). Cell cycle was analysed using a FACS Calibur flow cytometer (BD Biosciences, San Jose, CA, USA). Fifteen thousand events were evaluated using the Cell Quest (BD) software.

### Luciferase Assay and constructs

The 3'-UTRs of miR-181a, and miR-152 target mRNAs were amplified by PCR from human genomic DNA using the following primer pairs: hCOL16A1-3'UTR-F 5'-GGCCTCTAGA CCCCACCTGCCTTTGGATG -3'; hCOL16A1-3'UTR-R 5'-GGCCTCTAGAGACTGAGTCTCATTA GTTGC -3'; hITGA5-3'UTR-F 5'-GGCCTCTAGAGT CCTCCCAATTTCAGACTC -3'; hITGA5-3'UTR-R 5'-GGCCTCTAGACTAGTTCTGGTCAGTGGGGG -3'. PCR fragments were restricted and ligated to a compatible XbaI-linearized pGL3Control vector (Promega). Cells were transfected with 100ng of pGL3 vectors, 12pmol of pre-miR or a scrambled sequence (Ambion), and10ng of *Renilla* luciferase pRL-CMVvector (Promega). Luciferase assays were then performed as described before [87].

### Adhesion Assay

96-well microplates were coated for 1h at 37°C with 100 μg/ml rat tail collagen I (BD, Franklin Lakes, NJ USA) and blocked for 1h at 37°C with PBS, 1% (W/V) BSA.96h after transfection 1.2 x104 cells were detached, seeded into 96-well culture plate and incubated for 15 minutes at 37°C/5% CO_2_. The adherent cells were washed twice with PBS, fixed in 4% paraformaldehyde for 15 minutes and stained with 0.1% crystal violet in 2% ethanol for 10 minutes. Excess dye was removed by washing with H_2_O, the violet stain was solubilized with 2% SDS and absorbance was measured at 540 nm with ELISA reader.

### Western Blotting

Total cell extracts were resolved on a SDS polyacrylamide gel, blotted on a Hybond P PVDF membrane (G&E Healthcare, UK). Membranes were blocked with PBST 5% non fat dry milk, incubated with primary antibodies for 2 h at room temperature, washed and hybridized for 1h at room temperature using the appropriate horseradish peroxidase-conjugated secondary antibody (rabbit and mouse, BioRad, Hercules, California, USA). Detection was performed with the ECL chemiluminescence kit (Perkin Elmer, Waltham, Massachusetts, USA). The following antibodies were used: anti-p16 (Santa Cruz Biotechnology, California, USA; dilution 1:1000), anti-col16a1 (Proteintech, Chicago, USA; dilution 1:400), anti-itga5 (Santa Cruz Biotechnology, California, USA; dilution 1:500), anti-p53 (Santa Cruz Biotechnology, California, USA; dilution 1:500), anti-β actin (Sigma, St Louis, Minnesota, USA; dilution 1:5000).

### Bioinformatics

Analysis of miR-181A, and miR-152 target sites on COL16A1 and ITGA5 3'UTR were performed using the TargetScan 5.1 software available at http://www.targetscan.org/.

## Abbreviations

microRNA: miRNA or miR-
ITGA5: integrin α5
COL16A1: collagen XVI
ECM: extracellular matrix
HDFn: normal human dermal fibroblasts
SA: senescence associated
DEJ: derma-epidermal junctions
BrdU: 5-bromo-2'-deoxyuridine
